# LPS‐stimulated NF‐*κ*B p65 dynamic response marks the initiation of TNF expression and transition to IL‐10 expression in RAW 264.7 macrophages

**DOI:** 10.14814/phy2.13914

**Published:** 2018-11-13

**Authors:** Stuart Hobbs, Marinaliz Reynoso, Alyssa V. Geddis, Alexander Y. Mitrophanov, Ronald W. Matheny

**Affiliations:** ^1^ Military Performance Division U.S. Army Research Institute of Environmental Medicine Natick Massachusetts; ^2^ DoD Biotechnology High Performance Computing Software Applications Institute Telemedicine and Advanced Technology Research Center U.S. Army Medical Research and Materiel Command Ft. Detrick Maryland

**Keywords:** Inflammation, interleukin‐10, lipopolysaccharide, nf‐kappa B, TLR4, tumor necrosis factor

## Abstract

During injury and infection, inflammation is a response by macrophages to effect healing and repair. The kinetics of the responses of proinflammatory TNF *α*, anti‐inflammatory IL‐10, and inflammatory master regulator NF‐*κ*B elicited by lipopolysaccharide (LPS) may be critical determinants of the inflammatory response by macrophages; however, there is a lack of homogeneous kinetic data in this pathway. To address this gap, we used the RAW 264.7 macrophage cell line to define intracellular signaling kinetics and cytokine expression in cells treated with LPS for 15 min to 72 h. The abundance of I*κ*B*α* was maximally reduced 45‐min following LPS treatment, but expression increased at 10‐h, reaching a maximum at 16 h. NF‐*κ*B phosphorylation was significantly increased 45‐min following LPS treatment, maximal at 2‐h, and decreased to basal levels by 6‐h. Nuclear NF‐*κ*B expression was elevated 30‐min following LPS treatment, maximal by 45‐min, and returned to basal levels by 24‐h. Binding of nuclear NF‐*κ*B to consensus oligonucleotide sequences followed a similar pattern to that observed for p‐NF‐*κ*B, but lasted slightly longer. Following LPS treatment, TNF *α* mRNA expression began at 1‐h, was maximal at 6‐h, and decreased starting at 10‐h. TNF *α* protein secretion in conditioned growth medium began at 4‐h and was maximal by 16‐h. IL‐10 mRNA expression was induced by LPS at 10‐h, and was maximal at 16‐h. IL‐10 protein secretion was induced at 16‐h and was maximal at 24‐h. Our data reveal the temporal kinetics of pro‐ and anti‐inflammatory signaling events that may be important therapeutic targets for inflammatory diseases.

## Abstract 2

We have demonstrated that the activation state, nuclear residency, and DNA binding of NF‐κB mark the initiation of proinflammatory TNFα expression and also the switch to anti‐inflammatory IL‐10 expression in macrophages. We also demonstrate that inhibiting MYD88 expression inhibited the early‐mid TNF response and late IL‐10 response whereas inhibiting TBK1/IKKε (TRIF pathway) activity inhibited the responses through the entire LPS time‐course. These data will provide higher fidelity inputs for any computer model of this pathway, which is a strong interest for the inflammatory research field in order to identify targets for the therapeutic treatment of inflammatory diseases.

## Graphical abstract

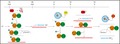

## Introduction

Inflammation is an orchestrated response by resident and recruited cell types, such as macrophages, to resolve infection and/or repair injury to tissues. Pathogen‐associated molecular patterns, such as lipopolysaccharide (LPS), and virulence factors are microbial inducers of inflammation. There is also an increasingly large library of endogenous inducers of inflammation that serve as signals of tissue injury or disruption of homeostasis (Medzhitov 2008; Kawai and Akira 2011). Tissue‐resident macrophages reacting to these signs of infection or injury exhibit a proinflammatory anti‐microbial M1 phenotype and secrete factors, such as tumor necrosis factor‐alpha (TNF*α*), that mediate additional inflammatory events. These events include responses in affected tissues and recruitment of additional macrophages from the pool of circulating monocytes. Macrophages then switch to an anti‐inflammatory M2 phenotype to complete the inflammatory response by secreting mediators, such as interleukin‐10 (IL‐10). These anti‐inflammatory factors inhibit the production of proinflammatory factors in order to prevent further tissue damage, and also secrete growth factors to stimulate wound healing through the activity of myofibroblasts (Arnold et al. 2007; Biswas and Mantovani 2010; Murray and Wynn 2011; Martinez and Gordon 2014; Shapouri‐Moghaddam et al. 2018).

The transcription factor NF‐*κ*B is a master regulator of the inflammatory response. NF‐*κ*B receives input from a variety of receptors and inflammatory inducers to promote context‐specific cellular responses that are appropriate to the stimulating inducer. NF‐*κ*B regulates the transcription of a wide variety of genes that are important to the inflammatory response including chemokines, cytokines, and adhesion molecules; and it also regulates genes that negatively regulate its own activity (Lawrence 2009; O'Dea and Hoffmann 2009; Oeckinghaus and Ghosh 2009; Mitchell et al. 2016). The significance and determinants of NF‐*κ*B dynamics in the inflammatory responses has been the subject of intense scrutiny in recent studies (Sung et al. 2014; Zambrano et al. 2014, 2016; Adamson et al. 2016; Sakai et al. 2017). One possible mechanism whereby NF‐*κ*B decodes multiple inputs into unique response outputs may be the duration of NF‐*κ*B activity. In mouse embryonic fibroblasts, TNF*α* stimulated a more transient NF‐*κ*B activation than that stimulated by LPS. This is due to TNF*α* stimulation being more sensitive to the negative regulator A20 than LPS stimulation because LPS induces expression of autocrine stimulatory cytokines that amplify IKK activation (Werner et al. 2005).

As a target and possible target of NF‐*κ*B regulation, respectively, TNF*α* and IL‐10 are important mediators of the immune response by macrophages (Medzhitov 2008; Saraiva and O'Garra 2010). Although TNF*α* is one of the primary early phase immune response proinflammatory cytokines, there is evidence from studies in TNF‐ and TNFR‐deficient mice that TNF can act to temper the inflammatory response and play a role in the repair process (Vieira et al. 1996; Marino et al. 1997; Reid and Li 2001). This protective role for TNF may be abrogated in diseases of chronic inflammation, and the later phase immune response anti‐inflammatory cytokine IL‐10 could be an important element in this role. IL‐10‐deficient mice produce enhanced amounts of TNF in response to LPS and they have an exaggerated endotoxin shock response, demonstrating an important feedback role for IL‐10 in TNF‐mediated inflammatory responses (Berg et al. 1995). Deregulation of this balance between these proinflammatory and anti‐inflammatory responses results in a variety of inflammatory diseases (Medzhitov 2008). A detailed knowledge of the kinetics of signal transduction events that couple inflammation inducers to inflammatory mediators, and the temporal relationship between proinflammatory (TNF*α*) and anti‐inflammatory (IL‐10) mediators, will aid in understanding how these diseases arise and how to therapeutically treat them.

One study attempted to address this issue of temporal relationship between signal transduction pathways and cytokine response by creating a computational model of the kinetic responses of TNF*α* and IL‐10 in macrophages built from the signal transduction network that comprises the LPS signaling axis (Tomaiuolo et al. 2016). One limitation to this study was fragmentation of the parameterizing and validating data; some of the data harvested from the published literature for the signal transduction and cytokine responses were from bone marrow derived macrophages (BMDMs) and others from the RAW 264.7 macrophage cell line utilizing different concentrations of LPS. A recent study describing the response of TNF*α* to LPS has demonstrated this may be a significant issue because of differences in the activation states of mitogen‐activated protein kinases (MAPKs) in BMDMs and the RAW macrophages cell line (Gottschalk et al. 2016). Furthermore, even though there have been studies describing some of these kinetic responses, the data have resulted from landmark legacy studies with less quantitative analytical methods or had abbreviated durations of observation (Virca et al. 1989; Sweet and Hume 1996; Baer et al. 1998; Lichtman et al. 1998; Raabe et al. 1998). We hypothesized that an analysis of the LPS‐stimulated response with less variable experimental conditions and a wider window of observation would more accurately define connections between components of this response. Therefore, the purpose of our study was to document a detailed kinetic study of the LPS‐I*κ*B*α*‐NF‐*κ*B signaling axis and its coupling to TNF*α* and IL‐10 cytokine production from a single macrophage cell model system over an extensive time‐scale of LPS treatment.

Here, we demonstrate that the activation state, nuclear residency, and DNA binding of NF‐*κ*B are markers for the initiation and decline of the TNF response and the switch to the IL‐10 response stimulated by LPS, and that an abbreviated inflammatory response fails to elicit an anti‐inflammatory response. We also demonstrate that the TLR4 signaling adapter MYD88 regulates the early‐mid TNF*α* response and late IL‐10 response whereas the TLR4‐coupled pathway components TBK1/IKK*ε* are required for the full response of TNF*α* and IL‐10 throughout the LPS time‐course.

## Materials and Methods

### Cell line and reagents

The RAW 264.7 macrophage cell line (Ralph and Nakoinz 1977; Raschke et al. 1978) was purchased from American Type Culture Collection (ATCC, Manassas, VA). Dulbecco's modified Eagle's medium (DMEM, containing high glucose, 110 mg/L sodium pyruvate, and L‐Glutamine), phosphate buffered saline (PBS), fetal bovine serum (FBS), penicillin‐streptomycin (P/S), Halt™ protease & phosphatase inhibitor cocktail (100X), TRIzol^®^ reagent, high capacity cDNA reverse transcription kit, TaqMan^®^ universal PCR master mix (no AMPErase^®^ UNG), TNF TaqMan^®^ gene expression primer/probe set (Mm00443258_m1), and IL‐10 TaqMan^®^ gene expression primer/probe set (Mm01288386_m1) were purchased from ThermoFisher Scientific (Waltham, MA). Albumin solution from bovine serum (BSA) was purchased from Sigma‐Aldrich (St. Louis, MO). For Western immunoblotting, monoclonal p‐NF‐*κ*B (3033S/AB_331284), total NF‐*κ*B (8242S/AB_10859369), monoclonal Vinculin (13901S), and monoclonal total I*κ*B*α* (4812S/AB_10694416) antibodies were purchased from Cell Signaling (Danvers, MA). Cell Lysis buffer (10X) and LPS were also purchased from Cell Signaling. Western immunoblotting monoclonal antibody for TATA‐binding protein (TBP, ab125009/AB_10972354) was purchased from Abcam (Cambridge, MA). Mouse TNF‐alpha Quantikine^®^ ELISA kits and Mouse IL‐10 Quantikine^®^ ELISA kits were purchased from R&D Systems (Minneapolis, MN). Nuclear extraction kits and TransAM™ NF‐*κ*B p65/p50/p52 transcription factor assay kits were purchased from Active Motif (Carlsbad, CA). TBK1/IKK*ε* inhibitor BX795 was purchased from Inivogen (San Deigo, CA). Optimem (1x), Lipofectamine RNAiMAX, Silencer Negative Control No. 1 siRNA, and Silencer Select Myd88 siRNA (s70236) were purchased from ThermoFisher Scientific (Waltham, MA).

### Cell culture

RAW 264.7 macrophage cells were grown in DMEM containing 10% FBS and penicillin/streptomycin (P/S) at 37°C in 5% CO_2_. All experiments were done within 10 passages after the cells being thawed. Experiments requiring whole cell lysis were seeded on six‐well dishes at 12 × 10^4^ cells/mL in 4 mL of media. Experiments requiring media collections or harvesting of RNA extractions were seeded on 35 mm dishes at 12 × 10^4^ cells/mL in 3.4 mL of media. Experiments requiring nuclear‐cytoplasmic extractions were seeded on 100 mm dishes at 17.2 × 10^4^ cells/mL in 16 mL of media. Twenty‐four hours after seeding, cells were starved for 16–24 h in DMEM containing 0.01% BSA and P/S (SF media). Cells were then treated with 10 ng/mL of LPS reconstituted in nuclease free‐water. Cells were treated with LPS in SF media. For small interfering RNA (siRNA) experiments, cells were reverse transfected with 3.125 nmol/L siRNA using the Lipofectamine RNAiMAX transfection reagent. Cells were incubated for 16–24 h with the siRNA‐RNAiMAX complexes. The cells were then starved in serum‐free cell growth medium supplemented with 0.01% BSA for 16 h and then treated accordingly. For BX795 inhibitor experiments, cells were pretreated either with DMSO vehicle control or with 1 *μ*mol/L BX795 dissolved in DMSO for 45 min before any treatment, as noted.

### Cell stimulation and lysis

Cells were treated with PBS (Mock) or treated with 10 ng/mL LPS and subsequently harvested for either whole cell lysate or nuclear‐ cytoplasmic fractionation at various. For whole cell lysate processing, media containing LPS was removed, cells were washed with ice‐cold PBS, and cells were incubated in 1× lysis buffer containing Halt™ protease & phosphatase inhibitor cocktail (1: 100 v/v) on ice for 5 min. Lysates were then clarified by centrifugation at 14,000*g* for 10‐min. For nuclear‐cytoplasmic fractionations, the nuclear extraction kit by Active Motif^®^ was utilized and performed according to the manufacturer's protocol. Protein lysate concentrations were determined by Bradford assay (Bradford 1976).

### Western blot analysis

Western immunoblotting was performed as described previously (Matheny et al. 2012). The analysis of whole cell lysates and nuclear‐cytoplasmic fractionations were performed using antibodies for total NF‐*κ*B, p‐NF‐*κ*B, Vinculin, TBP, and/or total I*κ*B*α*. Briefly, denatured lysates from Mock‐treated cells were loaded onto a PAGE gel and lysates from LPS‐treated cells were loaded onto a different PAGE gel, in parallel. The 15‐min Mock treatment lysate was loaded onto both PAGE gels in order to control for differences resulting from the processing of two separate Western blots, permitting comparison of the Mock‐treated and LPS‐treated samples. Western blot membranes were cut into strips containing the appropriate protein molecular weight spans in order to probe each membrane for multiple proteins. Band densities from the resulting X‐ray films were quantified using NIH Image J 1.60. For experiments using whole cell lysates, the band densities of each time‐point were normalized to a loading control (vinculin or total NF‐*κ*B). The results for each time‐point are expressed as the fold change in band density compared to the 15‐min Mock treatment.

### p65 NF‐*κ*B transcription factor binding assay

Cells were either Mock‐treated or LPS‐treated for varying durations and cytoplasmic and nuclear cell lysates were fractionated using the TransAM NF‐*κ*B kit by Active Motif, according to the manufacturer's instructions. Nuclear lysates (20 *μ*g protein/sample) were then assayed for NF‐*κ*B that bound to immobilized double‐stranded DNA oligonucleotides containing NF‐*κ*B consensus sequences, using the 96‐well TransAM NF‐*κ*B ELISA kit according to the manufacturer's instructions. An active NF‐*κ*B standard for the assay was generated by stimulating RAW 264.7 macrophage cells with 10 ng/mL of LPS for 4 h and the harvesting nuclear lysate. This active NF‐*κ*B nuclear lysate standard was serially diluted to generate a standard curve for the TransAM NF‐*κ*B ELISA kit for each data set. The absorbance of each well was read using the Dynex Technologies DS2^®^ Automated ELISA System.

### RNA extraction and qPCR analysis

RNA extraction was performed using TRIzol^®^ reagent by ThermoFisher according to the manufacturer's instructions for processing adherent cells. cDNA was generated from each RNA sample using the high capacity cDNA reverse transcription kit from ThermoFisher and was performed according to manufacturer's protocol. Real‐time PCR was performed exactly as described previously (Matheny et al. 2015) using TaqMan primers/probes from ThermoFisher specific for IL‐10 (mm01288386_m1) and TNF*α* (Mm00443258_m1). Measurements were taken in the exponential phase when fluorescence exceeded the threshold level of detection determined by the software. Relative input mRNA levels were determined using the cycle threshold (2^−ΔΔCT^) method. The results for each time‐point are expressed as the fold change in normalized expression compared to the 15‐min Mock treatment.

### Conditioned growth media collection and cytokine ELISA

At each time point post‐LPS treatment, RNA extractions and media collections were performed simultaneously. Conditioned growth media was harvested from cell cultures and clarified by centrifugation and frozen until processing. For the 6 h media change experiment, conditioned media 6 h post‐LPS treatment was replaced with fresh SF media. The conditioned media following the media change at 6 h was then harvested at 16, 24, 48, and 72 h after the initial LPS treatment. Controls for each time point included untreated cells without a media change at 6 h, untreated cells with a media change at 6 h, and LPS treated cells without a media change at 6 h post‐LPS treatment. A separate 6 hour LPS treatment and 6 hour control were also harvested for conditioned media and served as additional controls. RNA extractions were harvested simultaneously with media collections for all plates at each time point.

Aliquots from each sample were thawed and brought to room temperature. TNF*α* and IL‐10 ELISAs were performed on separate media aliquots of each sample. Some samples were diluted in SF media to allow absorbance readings to be within the linear range established with purified standards (included in the kit). All values provided in this paper are the undiluted concentrations for either IL‐10 or TNF*α*. For siRNA experiments, cytokine concentrations were normalized to cell lysate protein concentration to control for any siRNA toxicity. Assays were performed according to the protocols provided in each respective kit and were performed using the Dynex Technologies DS2^®^ Automated ELISA System (Chantilly, VA).

### Statistical analysis

All data are expressed as the mean ± SD, representative of 3–5 independently conducted experiments. The data for all figures were statistically analyzed with GraphPad Prism 5 software using a two‐way ANOVA with a Bonferroni multiple comparison post‐test. A value of *P *< 0.05 was considered significantly different.

## Results

### LPS stimulates nearly simultaneous I*κ*B*α* degradation, NF‐*κ*B phosphorylation, and NF‐*κ*B nuclear translocation

**Figure 1:**
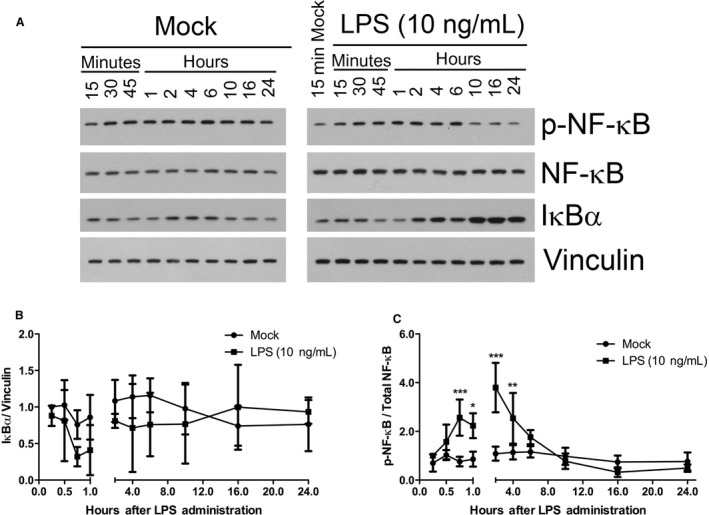
LPS stimulation of NF‐*κ*B phosphorylation and I*κ*B*α* degradation. (A) Western blot analysis of whole cell lysates for phospho‐NF‐*κ*B, total NF‐*κ*B, total I*κ*B*α*, and Vinculin. RAW 264.7 cells were either Mock‐treated or treated with LPS (10 ng/mL) for the indicated durations. Results are representative of 4–5 independent experiments. Normalized quantification of (B) total I*κ*B*α* and (C) p‐NF‐*κ*B expression levels from Western blot films in (A). X‐ray films were scanned electronically for protein band density quantification. (*P < 0.05; **P < 0.01; ***P < 0.001; Mock‐treated versus LPS‐treated; *N* = 4–5).

After determining that 10 ng/mL of LPS maximally stimulated phosphorylation I*κ*B*α* and its degradation (data not shown), we first investigated the kinetics of the LPS‐TNF*α* signaling axis by observing degradation of I*κ*B*α*, as well as NF‐*κ*B phosphorylation and nuclear translocation, in the RAW 264.7 macrophage cell line in response to durations of LPS stimulation ranging between 15 min and 24 h. LPS treatment induced a decrease in the expression of I*κ*B*α* in whole cell lysates (Fig. 1A and B) at 45‐min (0.32‐fold) and 1‐h (0.41‐fold) after LPS treatment. However, there was considerable experiment‐to‐experiment variability in I*κ*B*α* expression as assessed by Western blot, and as a result the differences in I*κ*B*α* expression between Mock‐treated and LPS‐treated RAW 264.7 cells were not significant at any of the time points tested. Despite this observed lack of significant degradation of I*κ*B*α* in whole cell lysates, LPS did significantly stimulate (2.6‐fold) activating phosphorylation of Serine 536 on NF‐*κ*B p65 (Fig. 1A and C) starting at 45‐min, and this reached a peak mean stimulation (3.8‐fold) at 2 h. By 6‐h, NF‐*κ*B phosphorylation had decreased to near Mock‐treated levels. There was some basal level of NF‐*κ*B phosphorylation in Mock‐treated cells, suggesting background constitutive activity of the pathway components in this macrophage cell line.

**Figure 2:**
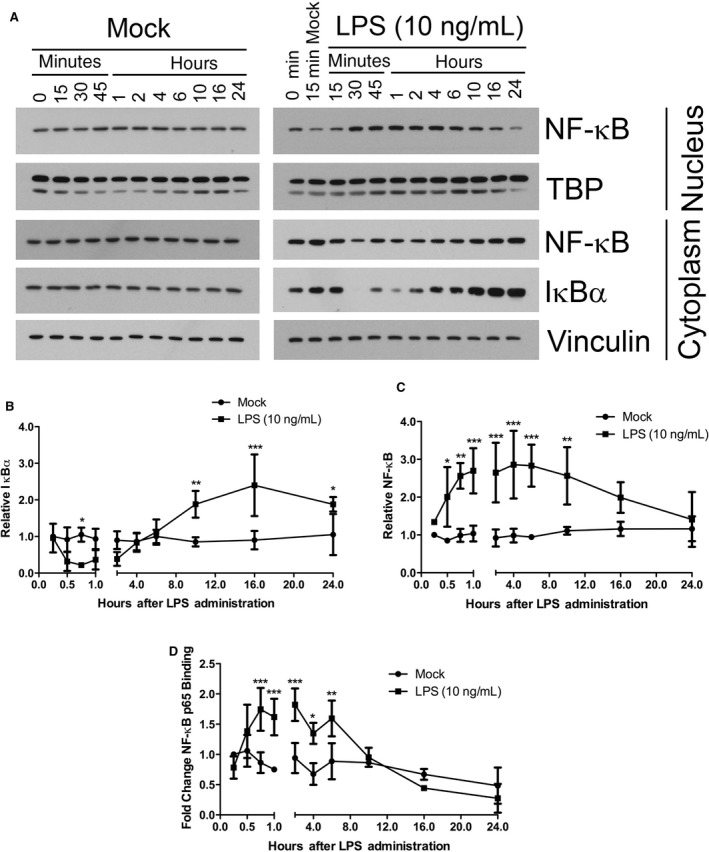
LPS stimulation of NF‐*κ*B nuclear translocation, cytosolic I*κ*B*α* degradation, and nuclear NF‐*κ*B p65 oligonucleotide binding. (A) Western blot analysis of fractionated nuclear and cytoplasmic lysates for total NF‐*κ*B, TATA‐binding protein, total I*κ*B*α*, and Vinculin. RAW 264.7 cells were either Mock‐treated or treated with LPS (10 ng/mL) for the indicated durations. Tata‐Binding Protein (TBP) is expressed in the nucleus and acted as a loading control for the nuclear lysates. Results are representative of 3–4 of independent experiments. Quantification of (B) cytoplasmic total I*κ*B*α* and (C) nuclear NF‐*κ*B from the Western blot films in A. X‐ray films were scanned electronically for band density quantification. (D) Quantification of nuclear p65 NF‐*κ*B binding to exogenous consensus DNA oligonucleotides. Nuclear lysates were assayed for transcriptionally active, nuclear NF‐*κ*B using the p65 NF‐*κ*B TransAM ELISA assay. (*P < 0.05; **P < 0.01; ***P < 0.001; Mock‐treated versus LPS‐treated; *N* = 3–4).

To clarify any effect by LPS on I*κ*B*α* and to further analyze the kinetics of subsequent nuclear translocation of NF‐*κ*B, we partitioned cell lysates into cytoplasmic and nuclear fractions following LPS stimulation. Fractionation of lysates did indeed decrease the apparent variability in I*κ*B*α* expression assayed by Western blot analysis. In the cytoplasmic fraction of cell lysates, the mean expression of I*κ*B*α* decreased sharply (0.32‐fold) at 30‐min after LPS treatment, but it was not significantly different from that of Mock‐treatment (Fig. 2A and B). However, at 45‐min LPS stimulated a decrease in I*κ*B*α* expression (0.22‐fold) that was maximal and significant compared with Mock‐treated cells. The mean I*κ*B*α* expression began increasing at 1‐h (0.36‐fold) and reached Mock‐treated mean levels at 4‐h.

The degradation of I*κ*B*α* and release of NF‐*κ*B results in the unmasking of a nuclear localization signal in NF‐*κ*B and subsequently permits its translocation to the nucleus, where it can act as a transcription factor. We thus analyzed nuclear lysate fractions for NF‐*κ*B expression to determine its translocation into the nucleus. LPS treatment induced a significant increase (2.0‐fold) in NF‐*κ*B expression starting at 30‐min compared with Mock treatment (Fig. 2A and C). The NF‐*κ*B translocation started to reach a plateau (2.7‐fold) after 45‐min and reached a peak mean value (2.9‐fold) at 4‐h. The increase in nuclear NF‐*κ*B expression continued to be significantly elevated (2.6‐fold) 10‐h after LPS treatment, after which it dropped to Mock‐treated levels. Starting at 10‐h after LPS treatment, I*κ*B*α* expression increased significantly (3.8‐fold) compared with Mock‐treated cells (Fig. 2B). This expression reached a peak mean value (5.6‐fold) at 16‐h and continued to be elevated (3.1‐fold) after 24‐h of LPS treatment. The peak expression of I*κ*B*α* at 16‐h was coincident with the drop in mean nuclear NF‐*κ*B expression at 16‐h that continued to decline at 24‐h. This overexpression of I*κ*B*α* at 10, 16, and 24 h in LPS‐treated cells contrasted sharply with the response of Mock‐treated cells whose I*κ*B*α* expression did not dramatically change during the entire measured time‐span.

### LPS‐stimulated NF‐*κ*B DNA binding overlaps with TNF*α* mRNA expression

Having investigated the kinetics of LPS‐stimulated phosphorylation and translocation of p65 NF‐*κ*B, we next sought to determine the kinetics of its ability to engage in regulation of transcription in the nucleus by assessing the binding of nuclear p65 NF‐*κ*B to promoter consensus sequences on immobilized DNA oligonucleotides. LPS stimulated a significant increase in NF‐*κ*B binding to consensus DNA sequences that started to plateau (1.7‐fold) at 45‐min and reached a peak mean value (1.8‐fold) at 2‐h (Fig. 2D). The increase in NF‐*κ*B binding to consensus sequences at 45‐min was coincident with the decreased cytoplasmic I*κ*B*α* expression, increased NF‐*κ*B phosphorylation, and increased NF‐*κ*B nuclear translocation at 45‐min. This extent of NF‐*κ*B binding to its regulatory sequences (1.6‐fold) persisted to 6‐h of LPS treatment and returned to Mock‐treated cell levels by 10‐h. The decrease in binding of NF‐*κ*B to consensus sequences at 10‐h was coincident with the increased I*κ*B*α* expression in cytoplasmic lysates (Fig. 2B).

**Figure 3:**
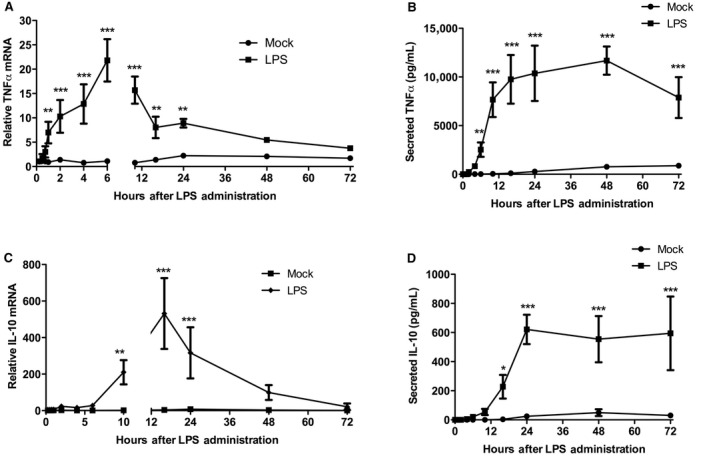
LPS stimulation of TNF *α* and IL‐10 mRNA production and protein secretion. (A) Quantitative PCR analysis of lysates for TNF *α* mRNA. (B) ELISA analysis of conditioned growth medium for secreted TNF *α* protein. (C) Quantitative PCR analysis of lysates for IL‐10 mRNA. (D) ELISA analysis of conditioned growth medium for secreted IL‐10 protein. RAW 264.7 cells were either Mock‐treated or treated with LPS (10 ng/mL) for 15 min, 30 min, 45 min, 1, 2, 4, 6, 10, 16, 24, 48, and 72 h, after which lysates and conditioned growth media was harvested. (**P < 0.001; ***P < 0.0001; Mock‐treated vs. LPS‐treated; *N* = 3–5).

To determine the functional significance of NF‐*κ*B binding of DNA, we detailed the kinetic response of its transcriptional target, TNF*α*. After 1‐h of LPS stimulation, TNF*α* mRNA expression increased (7.0‐fold) significantly (Fig. 3A). This continued to increase (21‐fold) to a maximal stimulation at 6‐h after LPS treatment. The mean value TNF*α* mRNA expression decreased (16‐fold) at 10‐h and continued to decrease, but was still significantly higher than that of Mock‐treated cells at 16 (6.6‐fold) and 24‐h (8.9‐fold). The decrease in TNF*α* mRNA at 10‐h was coincident with the cessation of NF‐*κ*B binding of consensus regulatory sequences (Fig. 2D) and with restored I*κ*B*α* expression (Fig. 2B). By 48 and 72‐h, LPS‐stimulated mean TNF*α* expression had decreased to nearly that of Mock‐treated cells.

We next assessed secreted TNF*α* protein concentrations following LPS stimulation. The secreted TNF*α* protein concentration was significantly elevated by LPS stimulation compared to Mock‐treatment by 6‐h (2.53 × 10^3^ pg/mL) and eventually reached a plateau in concentration by 16‐h (9.76 × 10^3^ pg/mL) and maximal peak mean value at 48‐h (11.7 × 10^3^ pg/mL) (Fig. 3B). The modest increase in secreted TNF*α* protein between 16 and 48‐h was consistent with the falling mRNA expression at 16‐h. By 72‐h of LPS treatment the mean secreted TNF*α* expression (7.88 × 10^3^ pg/mL) was still significantly elevated compared to Mock‐treated cells, but began to decrease.

### LPS stimulation of IL‐10 expression occurs subsequent to TNF*α* expression and cessation of NF‐*κ*B p65 activity, and requires full expression of TNF*α*

The decrease in the TNF*α* mRNA expression starting at 10‐h suggested the inhibitory action of the anti‐inflammatory IL‐10 response, so we next assessed the kinetics of this response. LPS stimulated a significant increase (210‐fold) in IL‐10 mRNA starting at 10‐h compared to Mock‐treatment (Fig. 3C), which coincided with secreted TNF*α* protein reaching its near‐plateau concentration (Fig. 3B). This also coincided with cessation of nuclear p65 NF‐*κ*B binding of consensus sequences (Fig. 2D). By 16‐h of LPS stimulation, IL‐10 mRNA had reached its peak expression level (531‐fold), and started to decrease by 24‐h (316‐fold) and decreased to near Mock‐treatment levels (99‐fold) by 48‐h. Interestingly, the peak expression of IL‐10 mRNA at 16‐h coincided with a cessation of nuclear p65 NF‐*κ*B binding of consensus sequences (Fig . 2D), near basal levels of nuclear NF‐*κ*B expression (Fig. 2C), and peak expression of I*κ*B*α* (Fig. 2B).

To substantiate the supposition that any negative feedback on TNF*α* mRNA expression was the result of IL‐10 and not some other LPS‐stimulated regulatory signal transduction, we measured secreted IL‐10 protein levels following LPS stimulation. Secreted IL‐10 was significantly increased (228 pg/mL) following 16‐h of LPS treatment (Fig. 3D). This increase coincided with the decline in TNF*α* mRNA expression (Fig. 3A). By 24‐h of LPS stimulation of IL‐10, protein secretion had reached its maximal mean concentration (621 pg/mL) and a response plateau (594 pg/mL) that was sustained as late as 72‐h. With respect to mRNA expression, the start of the IL‐10 response to LPS was delayed by 9‐h compared to the TNF response, and the peak level of IL‐10 was delayed by 10‐h compared to the TNF peak response. The delay for LPS‐stimulated protein secretion between IL‐10 and TNF*α* start and peak expression was very similar to that observed for the mRNA expression.

**Figure 4:**
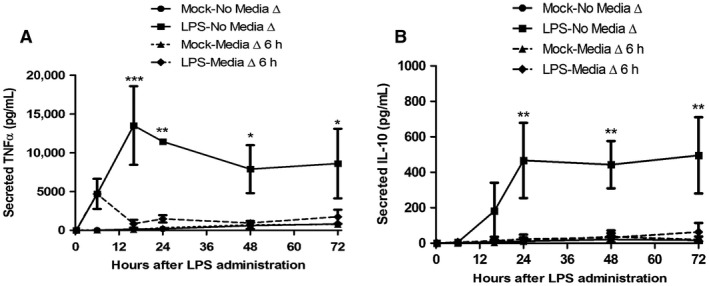
Production of TNF *α* and IL‐10 after chronic and acute LPS treatment. (A) TNF and (B) IL‐10 ELISA analysis of conditioned growth medium. RAW 264.7 cells were either Mock‐treated or treated with LPS (10 ng/mL) for the indicated durations in which either the serum‐free growth medium was not changed after treatment (No Media Δ), or the treated growth medium was replaced with un‐treated, serum‐free growth medium 6 h after initial treatment (Media Δ 6 h). Conditioned growth media was harvested 6, 16, 24, 48, and 72 h after initial treatment. (*P < 0.01; **P < 0.001; ***P < 0.0001, LPS – Media Δ vs. LPS – no Media Δ change; *N* = 3–5).

Because LPS stimulated its highest amount of TNF*α* mRNA at 6‐h that then decreased afterwards, we sought to determine whether this stimulated amount of TNF*α* mRNA would be sufficient to result in the observed TNF*α* secreted protein response at later time‐points, or whether chronic LPS treatment was required for the observed secreted protein induction. We also examined the response of IL‐10 secreted protein in this context to determine how tightly linked the pro‐inflammatory response was to the anti‐inflammatory response. In order to test this, cells were either treated with LPS as in previous experiments, or treated with LPS for 6‐h, after which the medium was removed and replaced with medium lacking LPS. Cells that had persistent LPS treatment continued to produced secreted TNF*α* (Fig. 4A) and IL‐10 (Fig. 4B) past 6‐h to an extent that had been observed in previous experiments (Fig. 3B and D). In contrast, when LPS treatment was withdrawn at 6‐h, cells expressed no secreted TNF*α* and IL‐10 protein above that expressed by Mock‐treated cells at 16‐h. Even after up to 72‐h neither TNF*α* nor IL‐10 expression was induced. The mean values for secreted TNF*α* were slightly different between the LPS treatments in Figures 3A and 4A; however, a statistical analysis determined that they were not significantly different.

### MYD88 expression is required for early‐mid TNF*α* expression and mid‐late IL‐10 expression induced by LPS

**Figure 5:**
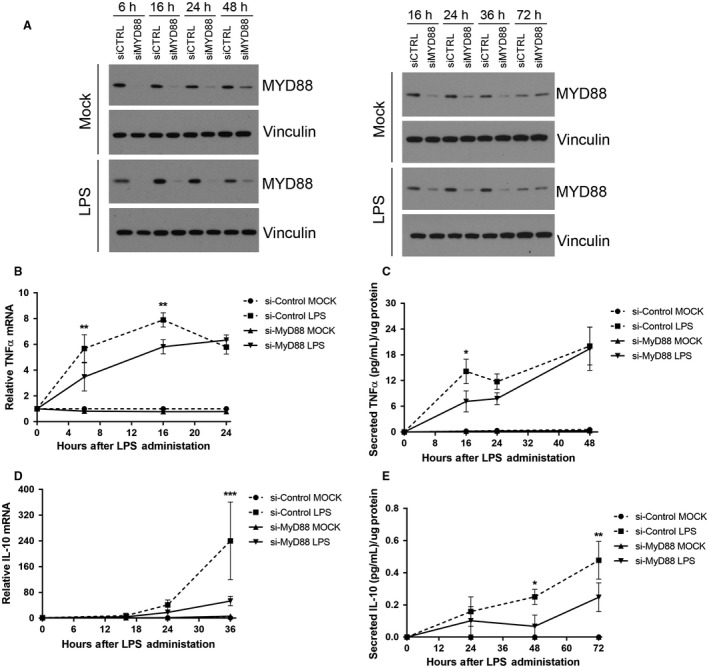
MYD88 siRNA transfection reduces early‐mid TNF *α* expression and late IL‐10 expression induced by LPS. (A) Western blot analysis of MYD88 expression following transfection with either control siRNA (siCTRL) or Myd88 siRNA (siMyd88). RAW 265.7 cells were either Mock‐treated or treated with LPS (10 ng/mL) for 6, 16, 24, 36, 48, and 72 h. (B) qPCR analysis of TNF *α* mRNA. Cells were transfected as indicated and either Mock‐treated or treated with LPS (10 ng/mL) for 6, 16, and 24 h. (C) TNF *α* ELISA analysis of conditioned growth medium. Cells were transfected as indicated, treated with LPS (10 ng/mL), and conditioned medium was harvested 16, 24, or 48 h later. (D) qPCR analysis of IL‐10 mRNA. Cells were transfected as indicated and either Mock‐treated or treated with LPS (10 ng/mL) for 6, 24, and 36 h. (E) IL‐10 ELISA analysis of conditioned growth medium. Cells were transfected as indicated, treated with LPS (10 ng/mL), and conditioned medium was harvest 16, 24, or 36 h later. (*P < 0.05; **P < 0.01; ***P < 0.001, siCTRL LPS vs. siMyd88 LPS; *N* = 3–5).

TLR4 couples to NF‐*κ*B through MYD88‐dependent and TRIF‐dependent pathways (Kawai and Akira 2007). To investigate the role of the TLR4 signaling adapter MYD88 in the observed kinetic responses to LPS, we used siRNA to inhibit MYD88 protein expression prior to stimulation with LPS. A reduction in MYD88 expression (Fig. 5A) resulted in a significant reduction in TNF mRNA of 39% and 26% early in the response at 6 and 16 h, respectively, after LPS treatment; however there was no affect at 24 h after LPS treatment (Fig. 5B). A significant reduction (49%) in secreted TNF*α* protein was evident only early at 16 h of LPS treatment (Fig. 5C). In contrast, reduction in MYD88 expression resulted in a significant IL‐10 mRNA decrease only later (78%) in the LPS‐induced response at 36 h (Fig. 5D). Similarly MYD88 expression knock‐down resulted in a significant decrease in secreted IL‐10, 73% and 48%, late in the response at 48 and 72 h, respectively, (Fig. 5E).

### TBK1/IKK*ε* is required for full TNF*α* and IL‐10 expression induced by LPS throughout the response

**Figure 6:**
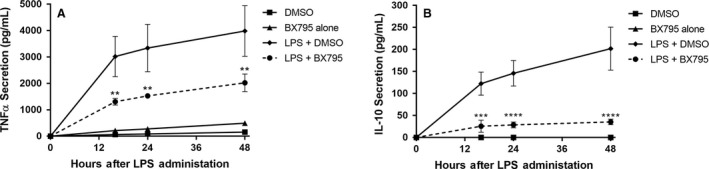
TBK1/IKK *ε* inhibitor BX795 blocks full expression of TNF *α* and IL‐10 stimulated by LPS. (A) TNF *α* and (B) IL‐10 ELISA analysis of conditioned growth medium. RAW 264.7 cells were pretreated with either DMSO or 1 *μ*mol/L BX795 and either Mock‐treated or treated with LPS (10 ng/mL), and conditioned growth media was harvested at 6, 16, 24, 48, and 72 h after initial treatment. (**P < 0.01; ***P < 0.001; LPS – DMSO vs. LPS – BX795; *N* = 3–5).

To investigate the role of the TRIF‐dependent signal transduction pathway components TBK1/IKK*ε* in the kinetic responses to LPS, we treated cells with LPS in the presence or absence of the TBK1/IKK*ε* inhibitor BX795. BX795 reduced induction of secreted TNF*α* protein by 57%, 54%, and 49% at 16, 24, and 48 h after LPS treatment, respectively, (Fig. 6A). BX795 reduced secretion of IL‐10 protein expression by 79%, 80%, and 82% at 16, 24, and 48 h, respectively, following LPS treatment (Fig. 6B).

## Discussion

**Figure 7:**
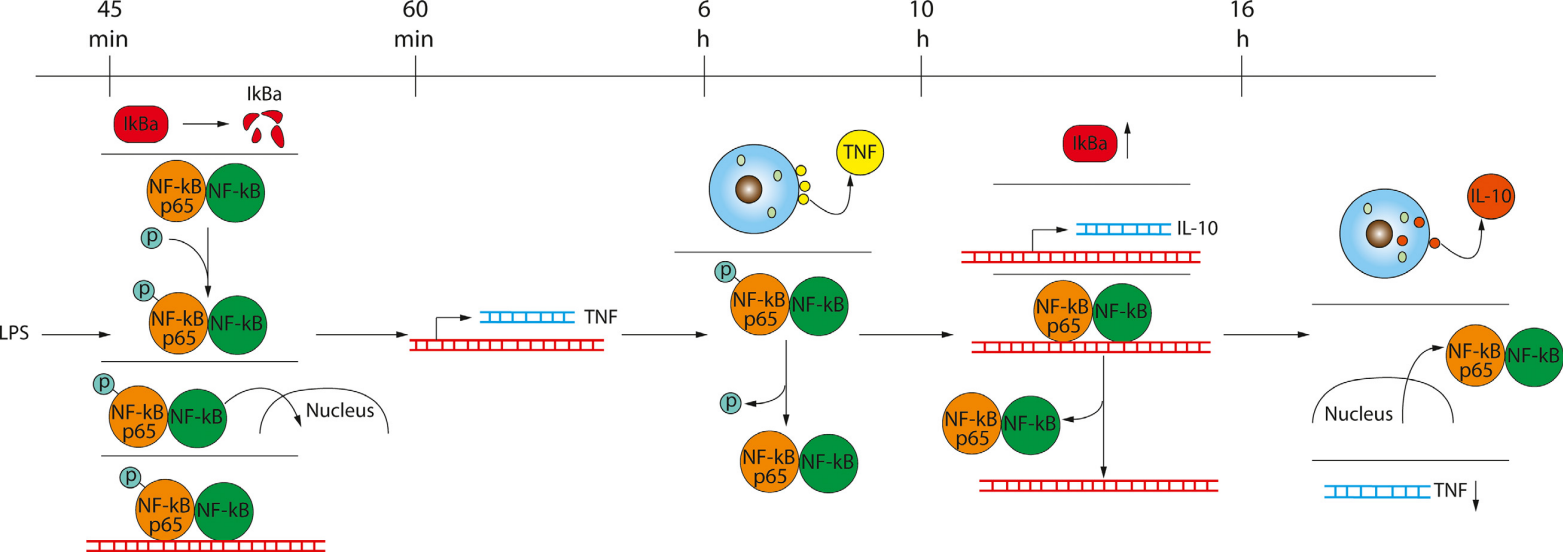
Kinetic timeline of LPS‐stimulated signal transduction through NF‐*κ*B and response of TNF and IL‐10. The top line indicates time elapsed after LPS treatment. Each column of events demarcated by a line indicates events that occurred at the noted time on the top line. DNA is indicated by red lines and mRNA is indicated by blue lines. The up arrow adjacent to I*κ*B*α* indicates an increase in protein expression and the down arrow adjacent to the TNF mRNA indicates a decrease in expression.

In this study, we resolved some of the temporal connections between the pro‐inflammatory TNF*α* expression, anti‐inflammatory IL‐10 expression, and I*κ*B*α* and NF‐*κ*B signaling event responses elicited by LPS in the RAW 264.7 macrophage cell line (Fig. 7). This is, to the best of our knowledge, the first kinetic study of the LPS‐I*κ*B*α*‐NF‐*κ*B signaling axis and TNF*α* and IL‐10 response using contemporary analytical methods in a single cell type over an extended time course up to 72 h. Establishing the coordinate responses of these signaling events now permits a more precise delineation of the relationships between these signaling respondents.

In general, the LPS‐NF‐*κ*B signaling axis was noticeably active within 45‐min of LPS treatment, in which there was degradation of I*κ*B*α*, phosphorylation of NF‐*κ*B, and binding of NF‐*κ*B to consensus regulatory DNA sequences. This was followed quickly, within minutes, by increased TNF*α* mRNA expression, and within hours by TNF*α* protein secretion. In contrast, the response of increased IL‐10 mRNA expression and protein secretion was delayed several hours after NF‐*κ*B activation, and instead quickly followed inactivation of NF‐*κ*B and its exit from the nucleus. The appearance of secreted IL‐10 was then quickly followed by a reduction in TNF*α* mRNA expression. Finally, in the absence of a full proinflammatory TNF*α* response, there was no anti‐inflammatory IL‐10 response.

Our results are consistent with the computationally predicted temporal hierarchy of the LPS‐triggered inflammatory response in macrophages (Tomaiuolo et al. 2016). In that work, the computational kinetic model captured the one‐peaked shape of the TNF*α* mRNA time course, which reached its maximal level shortly after the maximal NF‐*κ*B activity was achieved and then decayed visibly slower than the NF‐*κ*B activity. The following TNF*α* protein expression activation and subsequent decay were even slower, which is in accord with the timescales characterizing the extracellular signaling mechanisms engaged in TNF*α* regulation (Tomaiuolo et al. 2016). The substantial delay in the IL‐10 protein expression onset was one of the most robust features of the computational model simulations. It is plausible that this robust delay is functionally significant and serves to allow sufficient time for the transition between the M1 (i.e., proinflammatory) and M2 (i.e., anti‐inflammatory) macrophage phenotypes. It is generally understood that these two phenotypes are characterized, respectively, by increased TNF*α* and IL‐10 expression. Consistent with this paradigm and with the computational model simulations (Tomaiuolo et al. 2016), the experimental results in the present study show that the TNF*α* level begins to decrease when no considerable reduction in the IL‐10 level can be detected. This is a reflection of the IL‐10 protein stability, because the IL‐10 mRNA decays within 48 h of LPS exposure. Interestingly, while the absolute timing of the distinct stages of the inflammatory response can vary between different studies (e.g., compare (Tomaiuolo et al. 2016) with the results of the present work), their relative activation and deactivation order is preserved. This suggests that this relative temporal order may be a typical feature characterizing the inflammatory response under different experimental conditions.

Whereas the transition of I*κ*B*α*‐bound, inactive NF‐*κ*B to active, nuclear NF‐*κ*B was very rapid, its deactivation and return to steady‐state activity was more extended, with longer durations between changes in each measure of NF‐*κ*B activity. Upon LPS treatment, I*κ*B*α* degradation, NF‐*κ*B phosphorylation, nuclear translocation, and binding of DNA consensus sequences were nearly simultaneous events. The small delay between NF‐*κ*B activation and induction of TNF mRNA expression may have been due to marshalling and assembly of additional transcriptional regulatory proteins on the TNF*α* gene promoter. During the activation of this pathway then, we speculate that the rate‐limiting steps that may be more critical to determining whether and to what extent the response to LPS is, are either upstream of or parallel to NF‐*κ*B activation. This could include events such as those involved in regulating the coupling of TLR4 to its intracellular adapters, eventual ubiquitination of I*κ*B*α*, or phosphorylation of I*κ*B*α* and/or NF‐*κ*B.

In contrast, the deactivation of NF‐*κ*B was more discretely staged. In the first stage, NF‐*κ*B phosphorylation decreased, but NF‐*κ*B was still binding DNA at a point when I*κ*B*α* expression had returned to its basal expression. NF‐*κ*B phosphorylation recruits transcriptional‐enhancing cofactors, so dephosphorylation by phosphatases such as WIP1 may reduce transcription promotion by NF‐*κ*B and permit I*κ*B*α* binding (Chew et al. 2009). This might explain our observation of a decline in TNF*α* mRNA expression shortly after a decrease in NF‐*κ*B phosphorylation. The continued p65 NF‐*κ*B‐DNA binding and nuclear residency may function to promote and/or stall IL‐10 transcription in this stage either by forming a recruiting complex for additional transcription factors at the IL‐10 promoter or by competing with p50 for binding to the IL‐10 promoter.

In the second stage, which did not occur until hours later when I*κ*B*α* was increasingly abundant, NF‐*κ*B binding of DNA ceased. The I*κ*B*α* overexpression event may be critical for this second stage since I*κ*B*α* binding to NF‐*κ*B disrupts the ability of NF‐*κ*B to bind promoter sequences by increasing the rate of dissociation of NF‐*κ*B from DNA. It is interesting to speculate that the pool of nuclear resident NF‐*κ*B that is not bound to DNA may be performing some function to delay IL‐10 transcription, such as by binding IL‐10 transcription factors to prevent their binding to the IL‐10 gene promoter. We did not determine whether this retained nuclear NF‐*κ*B is bound to I*κ*B*α* or whether it is free NF‐*κ*B. If it is free NF‐*κ*B, it would suggest that I*κ*B*α* might have a lower affinity for free NF‐*κ*B than it does for DNA‐bound NF‐*κ*B. If this pool of NF‐*κ*B is binding some other proteins in the nucleus, they may compete for binding of NF‐*κ*B to I*κ*B*α*, lowering their apparent affinity for each other.

In the final stage, NF‐*κ*B exited the nucleus, hours after cessation of DNA binding. It is not clear why there is such a delay between cessation of NF‐*κ*B DNA binding and its complete exit from the nucleus. If I*κ*B*α* does indeed have a lower affinity for NF‐*κ*B that is not bound to DNA, it may take more time for a sufficient accumulation of I*κ*B*α* to bind and evacuate this pool of NF‐*κ*B from the nucleus. Another possible explanation for the delay is that the machinery that carries out nuclear export of NF‐*κ*B has a limited processivity, or alternatively, some other unknown mechanism is retaining NF‐*κ*B in the nucleus. Additional experiments will be needed to distinguish between these possibilities. Overall, the staged inactivation of NF‐*κ*B may be part of a mechanism to modulate the duration of TNF*α* response and delay the initiation of the IL‐10 response.

At least one report suggests that NF‐*κ*B's role in TNF*α* expression in response to LPS may be to facilitate post‐induction maintenance of TNF*α* mRNA during a late phase response rather than directly regulate transcriptional initiation (Tsytsykova et al. 2007). This conclusion was based in part on the low affinity of recombinant, non‐phosphorylated p65/p50 NF‐*κ*B heterodimer for a putative NF‐*κ*B binding motif in the proximal promoter of the TNF*α* gene (Udalova et al. 1998; Kuprash et al. 1999; Tsytsykova et al. 2007). The temporal proximity and precedence of NF‐*κ*B's activation to induction of TNF*α* mRNA expression in our study suggests that NF‐*κ*B has a more likely early direct role in transcriptional initiation. Also, since LPS‐stimulated NF‐*κ*B p65 phosphorylation appeared to closely correlate to the initial TNF*α* mRNA response, it raises the possibility that phosphorylation of NF‐*κ*B p65, may increase its affinity for this promoter element, and allow it to control TNF transcription. In fact, there is evidence that phosphorylation of sites within p65 increase its DNA binding (Ryo et al. 2003; Wan and Lenardo 2009). Further experimentation will be needed to substantiate a direct transcriptional role for NF‐*κ*B based on our kinetic correlation between NF‐*κ*B activity and TNF*α* response.

**Figure 8:**
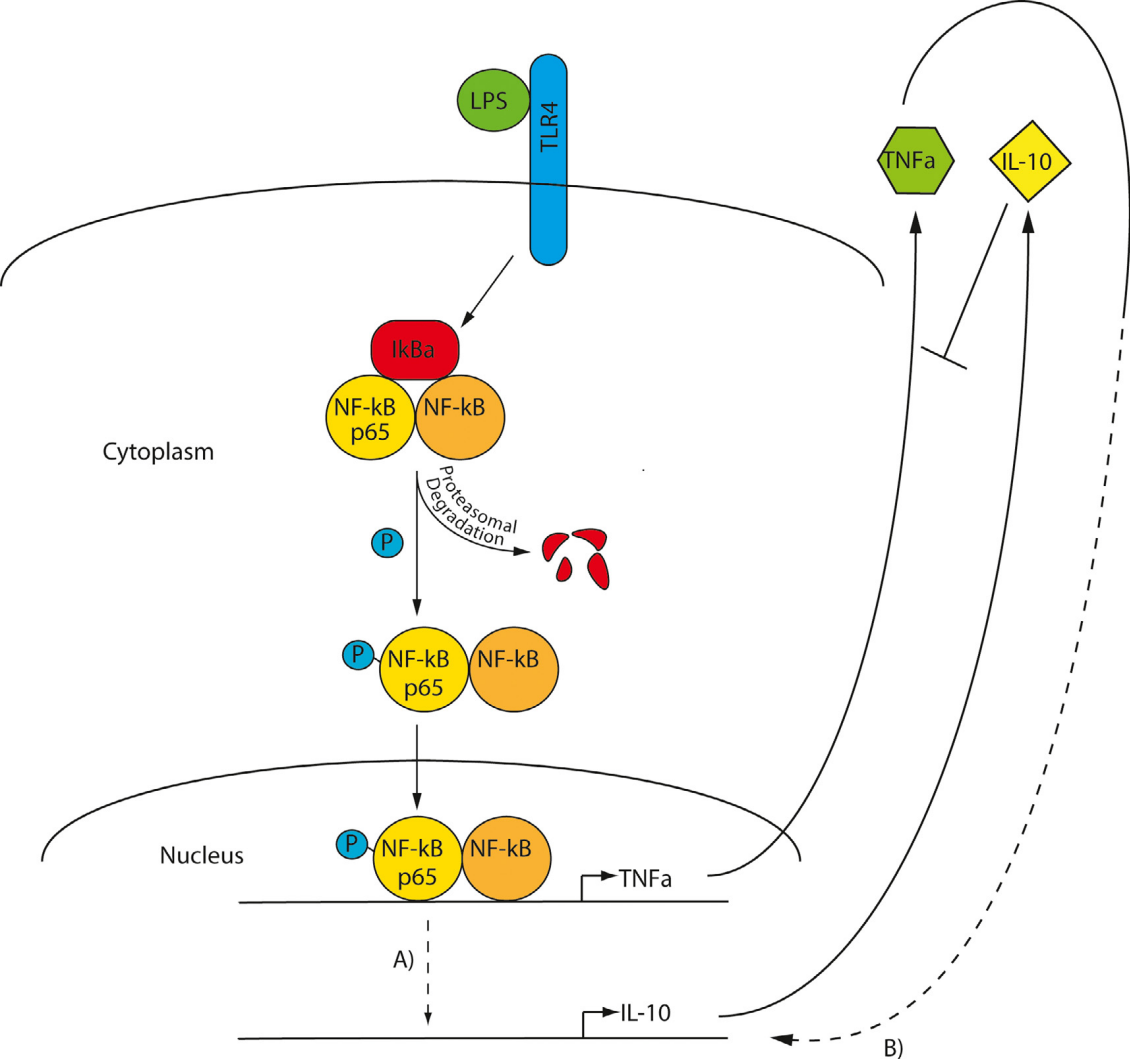
Schematic of the LPS‐I*κ*B*α*‐NF‐*κ*B signaling axis. LPS‐stimulated NF‐*κ*B may be regulating IL‐10 expression either by (A) the direct action of NF‐*κ*B through a repression or enhancing mechanism, or (B) indirectly by the signaling stimulated by TNF *α*.

Data from our and others’ studies suggest that NF‐*κ*B p65 may have direct, indirect, and repressor functions in the regulation of IL‐10 expression. A study identified an LPS‐responsive NF‐*κ*B p65 binding site far upstream of the IL‐10 transcription start site that functions as an enhancer for IL‐10 expression, (Iyer and Cheng 2012), and macrophages deficient in the upstream NF‐*κ*B regulator IKK2 had a profound decrease in IL‐10 expression in response to LPS, suggesting NF‐*κ*B is required for IL‐10 expression (Kanters et al. 2003). Therefore, it's possible in our study that NF‐*κ*B p65 may be acting directly (Fig. 8A) as a recruiting protein for additional transcription factors at the noted IL‐10 promoter site, during or after TNF expression, whereupon it dissociates from these sites after binding by I*κ*B*α* once the transcriptional enhancing complexes have assembled.

NF‐*κ*B may also act indirectly (Fig. 8B), providing a delay between the TNF*α* and IL‐10 response, by regulating transcription of a gene or genes whose activity stimulates IL‐10 expression. The delay between TNF*α* mRNA and IL‐10 mRNA expression was 1.5–1.8 times longer than the amount of delay between the start of observable mRNA expression and protein secretion for both TNF*α* and IL‐10. This suggests that there may be 2 sequential or slightly overlapping mRNA translation events that occur between induction of TNF*α* and IL‐10 mRNA expression, or that a single protein's expression has to rise to a threshold concentration before it triggers IL‐10 transcription. IFN‐*β* and IL‐27 are known regulators of IL‐10 that may fill this role (Iyer et al. 2010), but it is also possible that transcription/translation of the TNF*α* gene may account for this delay. Since TNF has been shown to stimulate IL‐10 expression in macrophages (Wanidworanun and Strober 1993; Huynh et al. 2016), it is possible that its effect on IL‐10 expression is delayed until TNF reaches a threshold concentration. Another piece of evidence for TNF regulating IL‐10 expression in macrophages is our final experiment (Fig. 4), which suggested that any active signaling instigated by TNF*α* at up to 6 h was not sufficient to trigger an anti‐inflammatory IL‐10 response, substantiating the idea of a threshold concentration of TNF*α* to induce IL‐10 expression. This experiment also demonstrated that macrophages have a mechanism for discontinuing the pro‐ and anti‐inflammatory response very quickly once an inflammatory inducer is eradicated.

Our data also compellingly suggest a possible IL‐10 transcriptional repressor function for NF‐*κ*B since IL‐10 expression occurs only during and after NF‐*κ*B p65 exit from the nucleus and after cessation of NF‐*κ*B p65 binding to promoter sequences (Fig. 7). This repression could be a mechanism for preventing a premature anti‐inflammatory response until the proinflammatory response had elicited the biological effects necessary for repair or resolution of infection.

Despite our observations tightly linking NF‐*κ*B signaling to TNF*α* expression, a recent article describing threshold control of TNF*α* expression by TLR signaling suggests that MAPK signaling is a “digital on‐off switch” for expression of inflammatory cytokines, whereas NF‐*κ*B functions as an “analog dial” to enable a continuum of phenotypic address and transcriptional readiness of inflammatory genes in response to low concentrations of microbial components (Gottschalk et al. 2016). The RAW 264.7 macrophage cell line we used in our study was described as having an aberrant TNF*α* response compared to that of primary bone marrow‐derived macrophages (BMDM) because of short‐circuited MAPK signaling in the RAW cells that permits TNF*α* expression to be responsive at lower concentrations of LPS. However, the response of I*κ*B*α* in the RAW cell line and BMDM was demonstrated to be very similar. Therefore, even though a limitation of the RAW cell model system may be its hypersensitivity to LPS with respect to the TNF*α* response, the observations we have made among NF‐*κ*B, TNF*α*, and IL‐10 response kinetics should be relevant, but perhaps more so at higher concentrations of microbial products in primary cells. Similar work on NF‐*κ*B dynamics in response to TNF in a migroglial cell line documented responses that were quite distinct from those that we observed with LPS, demonstrating that the cellular context established by the stimulus and the differential activities of signal transduction components such as MAPKs are critical determinants of the kinetic inflammatory response (Watters et al. 2002; Sheppard et al. 2011).

Recent studies have demonstrated that the TLR4 signaling adapters MYD88 and TRIF were both required for LPS induction of TNF*α*, but that they exerted distinct effects on NF‐*κ*B dynamics (Sakai et al. 2017). Our data suggest that the early LPS‐induced TNF*α* response may be more sensitive to altered MYD88 expression than later in the response. This is consistent with previous studies collectively demonstrating that MYD88 is involved in the early response, whereas the TRIF‐coupled pathway is involved in the later LPS response (Plociennikowska et al. 2015). The recent findings also demonstrated that in the absence of TRIF, TNF*α* transcription was ablated but NF‐*κ*B translocation still occurred, so the authors speculated that there is an unknown TRIF‐dependent mechanism that contributes to TNF*α* transcription (Sakai et al. 2017). TBK1/IKK*ε* are signaling molecules downstream of TRIF and are required for signaling that couples to IL‐10 expression (Biswas and Lopez‐Collazo 2009). In the absence of functional TBK1, mice were susceptible to LPS‐induced toxicity and their macrophages exhibited increased TNF*α* expression in response to LPS (Marchlik et al. 2010). In this context, our data showing an effect of the TBK1/IKK*ε* inhibitor throughout the LPS time‐course of TNF*α* response suggests that either IKK*ε* by itself or the combination of TBK1/IKK*ε* may be the TRIF‐dependent mechanism that couples LPS to induced TNF*α* expression.

We demonstrated that in contrast to the TNF*α* response, MYD88 was most critical to late IL‐10 expression in the LPS response. Overall, the muted IL‐10 response in the absence of full MYD88 expression is consistent with previous studies showing that MYD88 is required for the full induction of IL‐10 by LPS (Boonstra et al. 2006). If LPS‐induced IL‐10 expression is indeed driven to some extent by TNF*α*, then our data would suggest either that the early, 6–16 hour, TNF*α* response is most critical to the magnitude of the late IL‐10 response. This differential sensitivity of IL‐10 at different points in the kinetic response to MYD88 might suggest that there are distinct functions to the IL‐10 response at different phases that specify reactions tailored to different inflammatory inducers.

Finely tuned temporal control of the inflammatory response resulting from injury or infection is critical to resolution of the insult. If the pro‐inflammatory response endures too long, it can cause additional injury to the affected tissues. In the context of a bacterial infection that elicits a rapid, robust, or sustained inflammatory TNF response and an attenuated or absent anti‐inflammatory response, there is enhanced clearance of infection; however, there is also enhanced cellular apoptosis and septic shock (Tracey and Cerami 1994). In contrast, if the anti‐inflammatory response is too early, robust, or sustained, it will mute reparative proinflammatory effects or permit a state of persistent infection that will cause additional injury. Indeed, some pathogens have a survival program that shuts down host TNF production but still permits IL‐10 production in order to permit their host residency (Iyer and Cheng 2012). Our study provides a clearer understanding of the temporal connections between the pro‐ and anti‐inflammatory responses and their respective relationships to signaling coupled through NF‐*κ*B that could provide identity to signaling molecules or mechanisms that permit the previously noted instances of deregulated inflammatory response.

## Conflict of Interest

The authors have no conflicts of interest, financial or otherwise, to disclose.
